# Driving mechanism of farmers' green production behavior under normalization of COVID-19 prevention and control: A case study in China

**DOI:** 10.3389/fpubh.2022.826846

**Published:** 2022-09-15

**Authors:** Yun Teng, Boyuan Pang, Mei Zhang, Xiangyu Guo

**Affiliations:** ^1^College of Engineering, Northeast Agricultural University, Harbin, China; ^2^Postdoctoral Mobile Station of Agricultural and Forestry Economic Management, Northeast Agricultural University, Harbin, China; ^3^College of Economics and Management, Northeast Agricultural University, Harbin, China

**Keywords:** farmers' green production behavior, quality safety, agricultural products, public health, COVID-19

## Abstract

China's public health emergency COVID-19 has brought great challenges to food safety. Among them, the quality and safety of agricultural products under the normalization of the COVID-19 prevention and control has become a hot issue of general concern. This study attempts to reveal the driving factors and mechanisms of farmers' green production behavior. The empirical research by collecting 673 sample data shows that: individual characteristics of farmers, government guiding factors, an industrial organization promoting factors, and market adjustment factors have a positive driving effect on farmers' green production behavior. And farmers' green production behavior has a positive influence on the quality and safety of agricultural products. Farmers' green production behavior plays an intermediary role between the quality and safety of agricultural products and individual characteristics of farmers, government guidance factors, industrial organization promotion factors, and market regulation factors. The results of the study have guiding significance for ensuring the quality and safety of agricultural products, promoting ecological environmental protection, and sustainable agricultural development under the normalization of COVID-19 prevention and control.

## Introduction

It is crucial to ensure the quality and safety of agricultural products under the COVID-19. As a big agricultural country with a long history of development, China's traditional agricultural production concepts and production methods inevitably affect today's agricultural production. Improper use of agricultural machinery and excessive traditional irrigation patterns have resulted in soil compaction and changes in composition. The development of agricultural production methods that use large amounts of chemical fertilizers and pesticides has greatly weakened the stability and sustainable productivity of the agricultural ecosystem at the cost of huge ecological environment damage, resulting in increasingly serious environmental pollution problems. In particular, the COVID-19 pandemic has affected food safety in China. We should actively face the issue of quality and safety of agricultural products under the normalization of the COVID-19 epidemic, resolutely adhere to the bottom line of agricultural product quality and safety, and collaborate to win the battle against the epidemic.

Farmers' adoption of green production behaviors during the COVID-19 outbreak in China is the key to improving the quality and safety of agricultural products. Farmers' production behavior is a direct factor in determining the quality and safety of agricultural products (1–4); When farmers produce agricultural products, pesticides and fertilizers are used too casually, and some farmers even choose to use various illegal pesticides banned by the government. According to the testing of relevant state agencies, it was found that some farmers used sulfur to smoke their agricultural products, which made the agricultural products look better and facilitated sales or storage. Sulfur is highly toxic, and agricultural products smoked with sulfur are also highly toxic. Frequent consumption of sulfur-smoked agricultural products can lead to gastrointestinal disorders, abdominal pain, dizziness and other symptoms in mild cases, and in severe cases, it will lead to organ failure and even life-threatening. Therefore, the will of farmers plays a greater role in the production of agricultural products. Farmers' production behavior is an important factor affecting the quality and safety of agricultural products. Under the normalization of COVID-19 prevention and control, it is extremely important to actively promote farmers to adopt green production behaviors.

Establishing a multi-factor co-management mechanism under the normalization of COVID-19 prevention and control is an effective way for farmers to actively produce high-quality and safe agricultural products. The goal of comprehensively deepening the modernization of the national governance system and governance capacity can reflect China's transition from “national management” to “national government” and from “management thinking” to “governance thinking”. It is a way to provide useful inspiration to solve the problems of farmer production. At present, China's economic and social development concentrates in a complex environment with multiple centers and interdependence. Most of the agricultural production is dominated by a large number of small-scale and scattered farmers. The government cannot monitor and control farmers' production practices in all directions. The advantages of the market, government, industry and society are complementary. A multi-factor collaborative governance mechanism for farmers' green production behavior under the normalization of COVID-19 prevention and control has been constructed to promote farmers to adopt green production behavior to provide high-quality agricultural products. This is based on theoretical support and reflection from practice (2, 5).

Research on the quality and safety of agricultural products. In 1962, the book “Silent Spring” was published, which caused an uproar. The book proposed that excessive use of DDT pesticides has brought great harm to human health. Reducing the number of pesticides in food should be a way of marketing, just as it should be a moral imperative, which also raises great concern about the dangers of the overuse of pesticides. From the perspective of food science and technology, Professor King from the University of Washington, Howard, a British microbiologist, and Albrent, an American soil scientist, have improved the quality and safety of agricultural products through the cultivation of improved varieties, the improvement of soil environment, and the cultivation of pollution-free organic agricultural products. Other scholars have explored the causes and solutions of agricultural product quality and safety problems from the perspectives of economics, psychology, behavior, and marketing, including research on the external government supervision system for agricultural product quality and safety at the macro-level (6–8). To study the effect of pesticide application behavior of vegetable farmers on the quality and safety of agricultural products from the perspective of micro-subject behavior (1, 9, 10).

Research on pesticide application behavior of farmers. The research on pesticide application behavior of farmers from the micro-level is mainly based on the organization production school represented by Chayanov, the rational small farmer school represented by Theodor Schultz, and the classic farmer behavior theory represented by Huang Zongzhi. One school of thought believes that farmers' behavior is not to pursue the maximization of market profits, but to pursue the balance between consumer satisfaction and labor hardship; The other school believes that farmers' behavior is completely rational, and they make behavioral decisions with the goal of maximizing profits; Another school puts forward the more eclectic commodity smallholder theory, which holds that smallholders are both profit-seekers and subsistence producers, and their decision-making behavior is affected by both economic and non-economic factors (11, 12). Other scholars focus on the purpose, difference, reality, and economy of farmers' pesticide application behavior based on behavioral motivation theory, planned behavior theory, producer behavior theory, game theory, and information asymmetry theory. However, a unified conclusion has not yet been reached (13). Research on the influence of farmers' pesticide application behavior on the quality and safety of agricultural products. A survey report released by the World Health Organization confirmed that the phenomenon of excessive pesticide residues in vegetables is very common worldwide, and the direct factor is the illegal pesticide application behavior of vegetable farmers (14). It has been agreed that farmers' pesticide application behavior is a key factor affecting vegetable quality and safety. Controlling and standardizing vegetable farmers' pesticide application behavior is an important guarantee for vegetable quality and safety (15).

Research on fertilization behavior of farmers. There are economic factors, social factors, and their own internal factors that affect farmers' fertilization decision-making. Farmers' individual characteristics such as gender, age, planting experience, family characteristics, and education level will affect their green fertilization behavior (16–19). Farmers' fertilization is affected by land conditions and will choose fertilization conditions based on land conditions (20–22); Risk perception, green fertilization awareness, and green fertilization motivation have important effects on farmers' fertilization behavior (23, 24). Farmers' fertilization behavior is closely related to the government's attention (17), and the government should strengthen agricultural product quality and safety legislation, establish agricultural product quality and safety supervision agencies, and strictly supervise the behavior of agricultural producers (25). The government needs to reduce regulatory costs, increase agricultural subsidies, achieve full information sharing, increase farmers' additional benefits, and reduce production input costs to increase farmers' enthusiasm for green fertilization (26, 27). Sound high-quality agricultural product sales channels, supporting quality control standards and agricultural product quality traceability systems, and increasing consumer purchases and willingness to pay are favorable conditions that drive farmers to standardize fertilization (28). However, some scholars believe that government policies and regulations have a limited impact on farmers' fertilization behavior, and their standardization, sustainability, and availability need to be improved (14). Farmers provide agricultural products according to market demand, and farmers' production decisions conform to market rationality and are influenced by market regulation orientation (29, 30). The main factors affecting farmers' fertilization behavior are the proportion of farmers' income, the gains and losses of farmland protection, and the economic compensation for implementing protection policies (31). The implementation of subsidies for high-quality agricultural products, high sales prices, and market label certification are the key factors for farmers' green production, and the spillover income has a significant driving effect on farmers' standardized fertilization (32, 33). Awaken the food safety awareness of agricultural producers through social forces, give full play to the supervision role of social forces, and effectively urge producers to improve their fertilization behavior (34). Use online media and mainstream media to publicize and popularize scientific knowledge of agricultural product quality and safety, and promote farmers to standardize fertilization behavior (35).

To sum up, due to differences in political systems, social nature, and agricultural development environments between countries, the factors and driving means that lead to farmers' production behavior are different. While experts stress that implementing multiple drivers of farmers' production behavior in the future, their findings remain at the status quo description and conceptual stage. This study considers that ensuring the quality and safety of agricultural products is a major issue related to economic development and people's livelihood under the normalization of COVID-19 prevention and control. Using the ternary interaction theory, the hypothesis of multiple driving mechanisms of farmers' production behavior based on agricultural product quality and safety is proposed. A large amount of real data is collected through questionnaires, and the model is verified by the structural equation method, which provides scientific management and decision-making basis for solving the quality and safety of agricultural products in public health emergencies.

## Research methods and hypotheses

### Theoretical foundation

This paper proposes the hypothesis of multiple driving mechanisms of farmers' production behavior based on the quality and safety of agricultural products based on two theories of ternary interaction theory and psychological field theory.

Ternary Interaction Theory. The theory of ternary interaction is a famous learning theory proposed by the famous American psychologist Albert Bandura in the late 1970s by studying children's daily social learning. This finding mainly describes the inter-influence relationship between the three items of individual and environmental behavior. The three items are independent of each other, influence each other at the same time, and thus determine each other. The theory of ternary interaction is also widely used in education, business, decision leadership and medical treatment and other industries.

The interaction of individuals and behavior. People are the main body of behavior. People's attitude, consciousness, ability and cognition can influence and dominate people's behavior levels, so people's behaviors show differences. On the other hand, the result of the behavior gives feedback to the individual, which affects on the individual's consciousness, cognition, and subsequent behavior, thus forming a cycle between the individual and the behavior.

The interaction of the individual with the environment. As a member of the social environment, people do not exist in isolation, but are closely integrated with the surrounding environment. The material exchange, information transmission and energy conversion between people and the environment are constantly carried out, and the dynamic balance is always maintained, forming a dialectical unity relationship that is independent, restricting and interacting with each other, and has become an inseparable whole. Psychological factors such as personality, temperament, and values are not only affected by genetic factors, but also by the environment. Changes in the environment can stimulate people's psychology, affect people's emotions, and even disrupt people's normal activities. At the same time, human life and production activities continue to exert influence on the environment, thereby changing the nature and composition of the environment.

The interaction of behavior and environment. Behavior and the environment are interdependent. The environment not only affects the behavior of the individual, but also is changed by the behavior, that is, the development of the environment changes the behavior of the person, and the environment changes continuously with the exertion of human subjective initiative. When the environment is poor, there will be such a pattern: poor environment → people's psychology is adversely stimulated → disturbed people's actions → negative behaviors are generated. On the contrary, a good environment → stimulates people's favorable emotions → produces positive behaviors. The environment also plays an exemplary role in human behavior. When people are unable to make behavioral decisions, external demonstrative forces play a powerful role. This role is more apparent in authoritative environments, such as the leadership of a unit. Active work and active overtime work are likely to drive the entire company to form a good working atmosphere.

### Quality safety of agricultural products and farmers' green production behavior

The quality and safety of agricultural products are derived from the primary products of agriculture, that is, the reliability, usability, and intrinsic value of plants, animals, microorganisms, and their products obtained in agricultural activities. In general, the quality and safety of agricultural products mainly refer to the dual attributes of quality and safety of agricultural products. That is to say, agricultural products have both commodity attributes and value attributes. For example, the appearance, taste, packaging method, and nutritional content of agricultural products all meet certain standards. There are not only requirements for characteristics such as grades, specifications, and quality, but also requirements for the level of hazards to people and the environment. The farmers' green production behavior refers to whether the peasants choose to implement the standardized agricultural production techniques in the process of pursuing the maximization of their own interests, and produce a series of comprehensive behavioral responses in accordance with national standards for the production of qualified agricultural products. According to the positive and negative characteristics of human behavior, the farmers' production behavior can be divided into two categories. One of it is that farmers' green production behavior refers to the behavior of spontaneous production quality of qualified agricultural products. The other is that farmers' non-green production behavior refers to the passive production of quality qualified agricultural products or the unwillingness to produce quality. Farmers' green production behavior includes green pesticide application behavior and green fertilization behavior. Farmers can use cultivated land protection technology, precision fertilization technology, information technology, and biotechnology to precisely control the amount of fertilization, which can increase productivity while ensuring the quality of agricultural products (36). Arabian et al. (37) pointed out that farmers may suffer from cancer due to excessive pesticide exposure, and agricultural products with excessive pesticide application will also affect the health of end consumers. Many scholars' investigations and studies have found that farmers' non-green production behavior not only affects agricultural production, but also brings huge damage to consumers' health. People's long-term consumption of inferior agricultural products will lead to chronic poisoning and increase the incidence of cardiovascular and cerebrovascular diseases, Parkinson's, Alzheimer's, and other diseases. Scholars believe that to ensure the quality and safety of agricultural products, we must start from the source of production to prevent inferior agricultural products from appearing on the dining table, which affects the physical and mental health of consumers (38–40). Based on the above analysis, there are the following hypotheses:

Hypothesis H1: farmers' green production behavior has a positive effect on quality safety of agricultural products.

### Individual characteristics and farmers' green production behavior

Individual characteristics mainly involve gender, age, family population, education level, and years of farming. The gender differences in agricultural producers influence their behaviors in pesticide application (41). The factor of age is an important factor that distinguishes between pesticide application quantity and application frequency of agricultural producers. The older the agricultural producer is, the more likely it is to apply higher toxic pesticides (11, 42). Farmers with a higher level of education choose low concentration or standard concentration (15, 43). The annual household income, the number of households and pesticide of agricultural producers have different degrees of influence on the pesticide application behavior at different stages. With higher levels of education have a better understanding of agricultural product safety (44). In addition, the agricultural producers with the richer agricultural experience, the stronger their dependence on personal experience and the greater tendency to overuse pesticides based on past drug habits (45). Farmers with a high level of education can understand the importance of agricultural product safety issues and tend to avoid health threats through the use of fewer pesticides (4). Based on the above analysis, there are the following hypotheses:

Hypothesis H2: Individual characteristics factor have a driving effect on farmers' green production behavior.

### Government driving factors and farmers' green production behavior

The government driving factors mainly concern agricultural production laws and regulations, agricultural production standards, agricultural product subsidies, the retrospect of agricultural product quality, information sharing and symmetry, agricultural loans, propaganda and education, agricultural insurance, regulatory penalties, and detection of pesticide residues (7, 33, 46). Compared with developing countries, the actual use of pesticides in developed countries is not optimistic (47); it was found that the traceability of agricultural products can achieve the purpose of effectively reducing the use of pesticides. California government propaganda education can effectively reduce farmers' pesticide application (10). It is a good way for the government reducing regulatory costs, increasing agricultural subsidies, providing agricultural insurance, achieving adequate information, increasing penalties, increasing farmers' extra income, and reducing production input costs (45); This will increase government supervision of farmers' production practices effectiveness. Some scholars believe that the relevant government policies and regulations have limited impact on farmers' green production behavior. Furthermore, their standardization, sustainability and availability need to be further improved (12, 48). Based on the above analysis, there are the following hypotheses:

Hypothesis H3: The government driving factor has a driving effect on farmers' green production behavior.

### Industrial organizational driving factors and farmers' green production behavior

The organizational driving factors mainly involve technical training, acquisition agreements, unified supply of agricultural resources, unified defense control, brand strategy, recycling testing, and establishment of protective prices. Many scholars pointed out that whether farmers participate in industrial organizations has an important influence on the regulation of farmers' green production behavior (30, 33). However, most of the scholars who have studied the topic have targeted farmers' professional cooperation organizations (44). The effect of different industrial chain organizational models on farmers' green production behavior was significantly different. The cooperatives farmer households' model was superior to the association farmer household's model and higher than the enterprise farmer household's model. Chinese farmers' professional cooperatives which are transitioning from a loose type to a standardized one are gradually forming a normative organization with a corporate nature. Technical training of farmers' professional cooperatives, supply of agricultural resources, brand strategy, and recycling testing have important impact on farmers' green production behavior (13, 19, 49–51). Farmers' professional cooperatives are important carriers for small scale farmers to meet the challenge of modern market under the existing rural land system. Farmers' professional cooperatives play a decisive role in the farmers' production process (14, 52). Based on the above analysis, there are the following hypotheses:

Hypothesis H4: Industrial organization driving factors have driving effect on farmers' green production behavior.

### Market driving factors and farmers' green production behavior

Market driving factors are mainly related to the prices of agricultural products, prices of pesticides, market identification of safe agricultural products, consumer identification, and major uses of agricultural products (29, 53–55). The implementation of the price incentives for market identification certification in Switzerland successfully achieved the pesticide reduction target (8). The mechanism of market factors affecting farmers' production behavior (51). On the one hand, pesticide application reduces the loss of agricultural products output and benefit to farmers when the price of production changes overflows more than the price of pesticides. On the other hand, it can affect the quality and safety of agricultural products and thus affecting their prices, which bring a negative impact on the income. If markets can effectively distinguish the quality difference caused by the pesticide application amount, the greater the price drop of agricultural products caused by pesticide application, the more farmers tend to reduce the pesticides dosage (29). The market sales price of high-quality agricultural products is a key factor for farmers to adhere to sustainable production practices, and spillover benefits have a significant impact on farmers' green production behavior. Based on the above analysis, there are the following hypotheses:

Hypothesis H5: Market driving factors have a driving effect on farmers' green production behavior.

### Social driving factors and farmers' green production behavior

The social driving factors mainly concern social public opinion, mass supervision, media propaganda, internet communication, and third-party certification testing (28, 50). The solution to the quality and safety issues of agricultural products requires social forces consisting of propagation of “Agricultural Product Quality Safety Law” through Television stations, radio stations and newspapers and diffusion of agricultural product quality safety with the aid of online media and mainstream media, awakening the awareness of agricultural producers' food safety through social media, and prompting producers to improve their production behavior (29). Organic products and green product certification agencies and other social organizations can conduct third-party certification testing of agricultural product quality (51). In 2018, the Ministry of Agriculture logged a WeChat official account and establish joint propaganda and popular science mechanism to give full play to the positive role of social subjects in the process of governance of farmers' production behavior. Based on the above analysis, there are the following hypotheses:

Hypothesis H6: Social driving factors have a driving effect on farmers' green production behavior.

### Intermediary role of farmers' green production behavior

According to the ternary interaction theory, the environment affects individual psychological characteristics and individual psychological characteristics affect human behavior (24, 56) The technical training, the agricultural system supply, the brand strategy and the recovery test of the farmers' professional cooperative have an important influence on the farmer's production behavior. Farmers' professional cooperative is an important carrier to deal with the challenge of modern market under the existing rural land system, and the farmers' professional cooperative plays an important role in the process of farmers' production (9, 13). The influencing factors of pig farmers' safety production decision making shows that the farmers' professional cooperative is more closely connected with the pig farmers, and the management factors of farmers' professional cooperative has an impact on the safety production target and the safety production cognition of the pig farmers. Based on the above analysis, there are the following hypotheses:

Hypothesis H7: farmers' green production behavior plays an intermediary role between quality safety of agricultural products and driving factors.

### Hypothesized model construct

**Figure 1:**
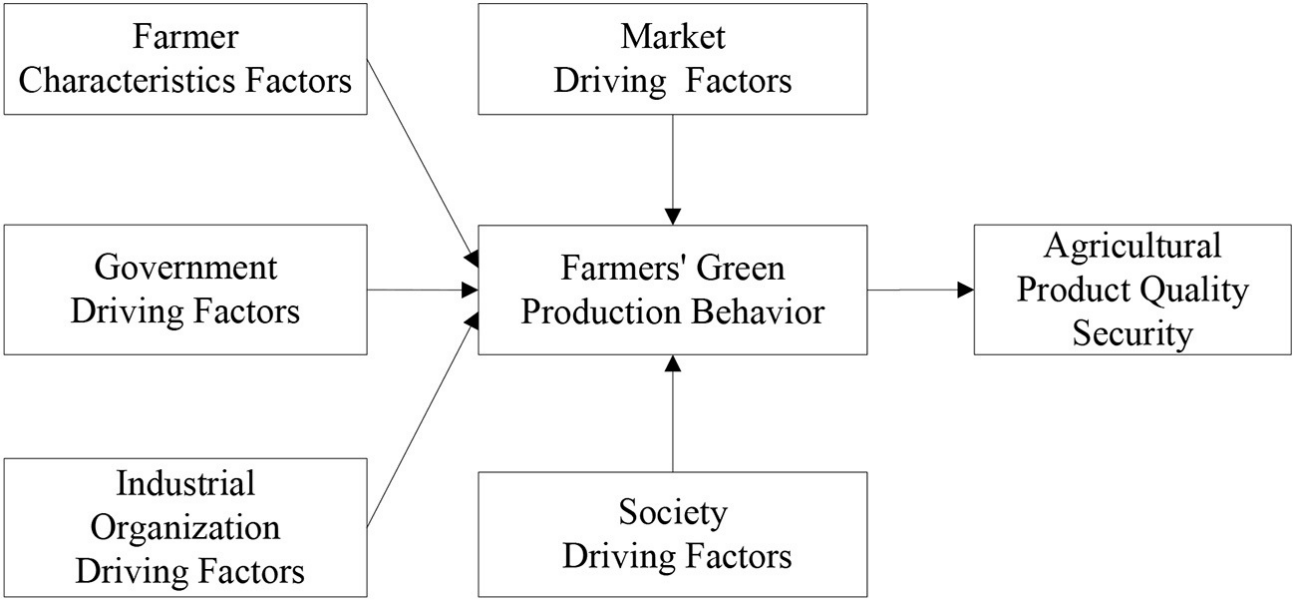
Hypothesized model.

Based on the above research hypothesis, the conceptual model of this study is shown in Figure 1.

## Research methodology

### Population and sample

This study is based on the quality and safety of agricultural products, and the entry point of agricultural producers' selection of farmers' production behavior based on the quality and safety of agricultural products. Through the questionnaire method, a combination of stratified design and random sampling was adopted, and typical agricultural provinces of Shandong, Henan and Sichuan were selected as representatives (Shandong has the highest arable land rate in China and is a major agricultural province in China, and its agricultural added value has long ranked first among all provinces in China. The cultivation of grain crops in Shandong includes typical crops such as winter wheat, corn, sweet potato, soybean, and rice; The area of arable land in Henan Province ranks second in China, and it is one of the three provinces and regions in China whose grain output exceeds the 30 million tons mark. Its wheat output ranks first in China, and it is one of the main soybean producing areas in China; Sichuan Province has a three-season farming system. The output of grain crops accounts for 65% of the total planting area. The main crops are rice, wheat, corn, potatoes, soybeans, etc., and the planting advantages are obvious). The field survey time was from January 2019 to June 2019. The population of Shandong Province was 101 million, the population of Henan Province was 99 million, and the population of Sichuan Province was 84 million. There are 51 prefecture-level city administrative districts under the jurisdiction of Shandong, Henan and Sichuan provinces as sampling cities (districts). Randomly sample 1–2 townships in each city (district), randomly sample 1 administrative village in each township, and randomly select about 10 farmers in each administrative village who have experience in fertilizing and applying pesticides. Seven hundred questionnaires were collected through face-to-face interviews, WeChat voice interviews, online questionnaires and mailings, among which 27 invalid questionnaires were screened out, and 673 valid questionnaires were obtained, with an effective rate of 96.1%. The specific survey results are shown in Table 1.

**Table 1:** Basic information sheet.

**Index**	**Indicator** **distribution**	**Frequency**	**Percentage/%**
Age	[20–30]	5	0.7
	[31–40]	91	13.5
	[41–50]	96	14.3
	[51–60]	233	34.6
	[61 and above]	248	36.9
Gender	male	409	60.8
	Female	264	39.2
Farming years	<10 years	91	13.5
	10–20 years	92	13.7
	21–30 years	190	28.2
	31–40 years	191	28.4
	More than 40 years	109	16.2
Education level	Did not go to school	257	38.2
	Primary school	297	44.1
	Junior high school	107	15.9
	High school	5	0.8
	University and above	7	1.0

### Survey instrument

The scale of this research: (1) Quality safety of agricultural products refer to the research results of Zhou Jiehong, Wang Jianhua and so on, the revised scale has 3 items (1r ate of eligible agricultural products, 2 quality level of agricultural products); (2) Farmers' green production behavior refer to the research results of Zhou Jiehong, Wang Jianhua and so on, and the revised scale includes 6 items (3 regulating production behavior, 3 regulating production awareness); (3) Farmer characteristics factors refer to the research results of Zhou Jiehong, Wang Haitao, Wang Jianhua, and the revised scale includes 5 items; (3 physiological characteristics, 2 plant features); (4) Government driving factors refer to the research results of Zhou Jiehong, Wang Haitao, Wang Jianhua, the revised scale has 6 items (3 government restraint factors, 3 government motivation factors); (5) Industrial organization driving factors refer to the research results of Zhou Jiehong, Wang Haitao, Wang Jianhua, the revised scale has 5 items (3 organization restraint factors, 2 organization motivation factors); (6) Market driving factors refer to the scale of the research results of Zhou Jiehong, Wang Haitao, Wang Jianhua, the revised scale includes 4 items (2 market restraint factors, 2 market motivation factors); (7) Social driving factors refer to the research results of Zhou Jiehong, Wang Haitao, Wang Jianhua, the revised scale has 5 items (2 social restraint factors, 3 social motivation factors) (51, 52). All scales were measured by Likert 1–5 scale, from 1 (totally disagree) to 5 (totally agree). The specific list is shown in Table 2.

**Table 2:** List.

**Driving factor**	**Category**	**Connotation**
Quality safety of agricultural products (QSAP)	Ate of eligible agricultural products	Nutritional quality and hygienic quality have passed national testing standards
	Quality level of agricultural products	Nutritional value of agricultural products
		Pesticide and fertilizer residue levels
Farmers' green production behavior (FGPB)	Regulating production behavior	The behavior of applying pesticides and fertilizers in accordance with the normative frequency
		The act of applying pesticides and fertilizers at safe intervals
		Measure the use of pesticides and fertilizers in accordance with norms
	Regulating production awareness	Awareness of applying non-toxic and low-toxic pesticides and fertilizers
		Awareness that excessive use of pesticides and fertilizers is harmful to people's health
		Awareness of the impact of pesticides and fertilizers on the environment
Farmer characteristics factors (FCF)	Physiological characteristics	age
		Education level
		marital status
	Plant features	Arable land planting area
		Planting cost (yuan/mu/year)
Government driving factors (GDF)	Government restraint factors	tax on agricultural producers
		Punish farmers for producing inferior agricultural products
		Agricultural product quality supervision
	Government motivation factors	Subsidies for high-quality agricultural products
		publicity and education
		Agricultural Production Loan Concessions
Industrial organization driving factors (IODF)	Organization restraint factors	Agricultural product quality inspection
		Uniform distribution of green pesticides and fertilizers
		Organizational management rules and regulations
	Organization motivation factors	Organizing unified training
		Incentives for producing high-quality agricultural products
Market driving factors (MDF)	Market restraint factors	Agricultural product quality market supervision
		Quality agricultural product market logo
	Market motivation factors	High quality and high price agricultural products
		Smooth sales channels for high-quality agricultural products
Social driving factors (SDF)	Social restraint factors	Third-party certification and testing
		public opinion
	Social motivation factors	People's recognition of high-quality agricultural products
		Online publicity of agricultural product quality and safety
		Consumer demand for high-quality agricultural products

### Data analysis

This research uses IBM SPSS Statistics Software 22 and LISREL10 software statistical analysis software to analyze the collected data. The data analysis mainly includes homologous deviation test, reliability and validity test, descriptive statistical analysis, hypothesis effect test.

## Research results

### Control and test of homologous deviation

Harmanda factor test showed that the first principal component was 20.4% when unroasted by Harmanda factor test, and the problem of homology is small and negligible.

### Test of reliability and validity

The reliability test mainly refers to Cronbachs'a coefficient. The Cronbachs' a coefficient of each scale in Table 3 is >0.7. The Kaiser-Mayer-Olkin (KMO) metric value is 0.854, which is >0.8, Bartlett's spherical test *P*-value is <0.01, indicating that the sample is suitable for factor analysis. The validity of the scale was tested from the content validity, convergence validity and discriminate validity (57, 58). The data showed that the factor loadings of each driving factor were all above 0.5, and the T values all reached a significant level, indicating that the data had high convergent validity. The correlation coefficient method is used to determine the degree of correlation between the driving factors, and to determine that the significant relationship between the two is significant. The specific verification results are shown in Table 3.

**Table 3:** The reliability and validity.

**Factor**	**cronbach's** **alpha**	**AVE**	**Composite** **reliability**	**Overall fitting index**
Quality safety of agricultural products (QSAP)	0.835	0.571	0.849	*χ^2^/df=2.23 RMSEA(0.048)* *IFI(0.90)CFI(0.91)GFI(0.95)* *TLI(0.991)*
Farmers' green production behavior (FGPB)	0.799	0.764	0.922	
Farmer characteristics factors (FCF)	0.812	0.667	0.904	
Government driving factors (GDF)	0.827	0.817	0.951	
Industrial organization driving factors (IODF)	0.784	0.711	0.930	
Market driving factors (MDF)	0.805	0.782	0.906	
Social driving factors (SDF)	0.859	0.763	0.894	

### Descriptive statistical analysis

Table 4 provides the statistics of mean value, standard deviation and variance. The main variables in the hypothesis are all relevant, as shown in Table 4.

**Table 4:** The descriptive and correlation analysis results.

**Factor**	**M**	**SD**	**QSAP**	**FGPB**	**FCF**	**GDF**	**IODF**	**MDF**	**SDF**
QSAP	2.246	0.590	1						
FGPB	3.228	0.420	0.523^**^	1					
FCF	4.258	1.096	0.431^**^	0.633^*^	1				
GDF	2.243	0.585	0.456^**^	0.717^**^	0.438	1			
IODF	2.312	0.443	0.531^**^	0.549^**^	0.214	0.289^*^	1		
MDF	3.243	0.416	0.355^**^	0.558^**^	0.062	0.455^*^	0.203	1	
SDF	3.132	0.494	0.597^**^	0.611^*^	0.183	0.216^*^	0.251	0.329^*^	1

* P < 0.05;

** P < 0.01; M represents mean; SD represents the standard deviation.

### Hypothesis effect test

The effect test on the main variable is shown in Table 5.

**Table 5:** The verification hypothesis effect.

	**A1**	**A2**	**A3**	**A4**	**A5**	**A6**	**A7**	**A8**	**A9**	**A10**	**A11**	**A12**	**A13**	**A14**	**A15**	**A16**	**A17**	**A18**	**A19**	**A20**
PC	−0.139^*^	−0.572^**^																		
PF	.		0.351^**^	−0.255^*^																
GRF					0.466^**^	0.158^*^														
GMF							0.703^**^	0.636^**^												
ORF									0.590^**^	0.377^*^										
OMF											0.408^**^	0.515^**^								
MRF													−0.493^**^	0.534^**^						
MMF															0.664^**^	0.479^**^				
SRF																	0.364^*^	0.379^*^		
SMF																			0.322^*^	0.296^**^
*R* ^2^	0.152	0.423	0.346	0.318	0.432	0.284	0.399	0.377	0.324	0.366	0.303	0.411	0.336	0.299	0.364	0.332	0.284	0.236	0.316	0.277
F	14.4^*^	38.9^**^	33.8^**^	35.6^**^	39.3^**^	27.1^**^	36.1^**^	28.9^**^	31.1^**^	37.0^**^	30.4^**^	33.2^**^	30.3^**^	33.8^**^	35.6^**^	30.3^**^	26.2^**^	33.1^**^	29.7^**^	30.2^**^
	**B1**	**B2**	**B3**	**B4**	**B5**	**B6**	**B7**	**B8**	**B9**	**B10**	**B11**	**B12**	**B13**	**B14**	**B15**	**B16**	**B17**	**B18**	**B19**	**B20**
PC	−0.114^*^	−0.293^**^																		
PF	.		0.203^**^	−0.132^*^																
GRF					0.361^**^	0.099^**^														
GMF							0.337^**^	0.245^**^												
ORF									0.199^**^	0.254^*^										
OMF											0.384^**^	0.315^**^								
MRF													−0.391^**^	0.244^**^						
MMF															0.363^**^	0.233^**^				
SRF																	0.164^*^	0.248^*^		
SMF																			0.244^*^	0.089^**^
RMB	−0.132^*^	−0.322^**^	0.388^**^	−0.300^**^	0.463^**^	0.684^**^	0.518^**^	0.574^**^	0.482^**^	0.463^**^	0.684^**^	0.467^**^	−0.571^**^	0.530^**^	0.637^**^	0.647^**^	0.211^*^	0.445^**^	0.546^**^	0.462^**^
RMA	−0.101^*^	−0.429^**^	0.461^**^	−0.411^**^	0.628^**^	0.678^**^	0.437^**^	0.635^**^	0.545^**^	0.500^**^	0.741^**^	0.743^**^	−0.851^**^	0.640^**^	0.477^**^	0.847^**^	0.346^*^	0.521^**^	0.466^**^	0.332^**^
*R* ^2^	0.235	0.324	0.357	0.372	0.393	0.352	0.351	0.342	0.364	0.343	0.352	0.435	0.397	0.243	0.378	0.356	0.135	0.436	0.334	0.345
F	16.2^*^	35.6^**^	33.6^**^	35.6^**^	38.6^**^	30.5^**^	38.4^**^	32.1^**^	37.4^**^	33.7^**^	35.7^**^	29.9^**^	38.9^**^	34.6^**^	37.9^**^	36.9^**^	30.9^*^	39.4^**^	30.4^**^	39.4^**^

N = 673;

** stands for p < 0.01;

* stands for p < 0.05.

Regression analysis of characteristics factors on farmers' green production behavior. In model A1 and A2, the regression coefficients of physiological characteristics for regulating production behaviors and regulating production awareness are, respectively (*β* = −0.139, *P* < 0.05) and (*β* = −0.572, *P* < 0.01). In model A3 and A4, the regression coefficients of plant features for regulating production behaviors and regulating production awareness are, respectively (*β* = 0.351, *P* < 0.01) and (*β* = −0.255, *P* < 0.05).

In model A5 and A6, the regression coefficients of government motivation factors for regulating production behaviors and regulating production awareness are respectively (*β* = 0.466, *P* < 0.01) and (*β* = 0.158, *P* < 0.05). In model A7 and A8, the regression coefficients of government restraint factors for regulating production behaviors and regulating production awareness are, respectively (*β* = 0.703, *P* < 0.01) and (*β* = 0.636, *P* < 0.01).

In model A9 and A10, the regression coefficients of organization restraint factors for regulating production behaviors and regulating production awareness are, respectively (*β* = 0.590, *P* < 0.01) and (*β* = 0.377, *P* < 0.05). In model A11 and A12, the regression coefficients of organization motivation factors for regulating production behaviors and regulating production awareness are, respectively (*β* = 0.408, *P* < 0.01) and (*β* = 0.515, *P* < 0.01).

In model A13 and A14, the regression coefficients of market restraint factors for regulating production behaviors and regulating production awareness are, respectively (*β* =-0.493, *P* < 0.01) and (*β* = 0.534, *P* < 0.01). In model A15 and A16, the regression coefficients of market motivation factors for regulating production behaviors and regulating production awareness are, respectively (*β* = 0.664, *P* < 0.01) and (*β* = 0.479, *P* < 0.01).

In model A17 and A18, the regression coefficients of social restraint factors for regulating production behaviors and regulating production awareness are, respectively (*β* = 0.364, *P* < 0.05) and (*β* = 0.379, *P* < 0.05). In model A19 and A20, the regression coefficients of social motivation factors for regulating production behaviors and regulating production awareness are, respectively (*β* = 0.322, *P* < 0.05) and (*β* = 0.296, *P* < 0.01). In summary, hypothesis H1, H2, H3, H4, and H6 are established.

**Figure 2:**
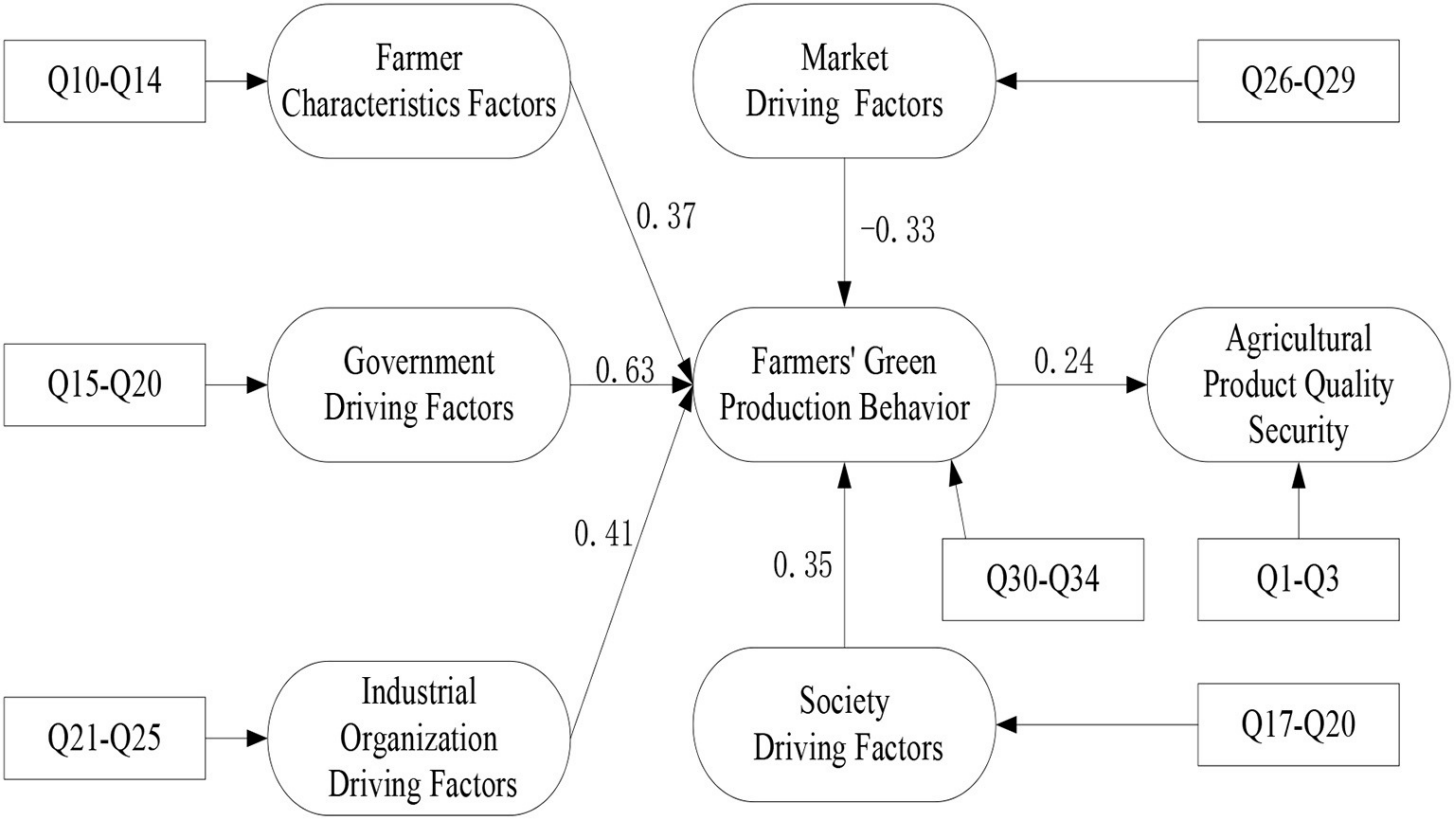
Mediation effect map.

Government driving factors and farmers' green production behavior do regression analysis on agricultural product quality security. After the model B5, B6 are added to the model A5, A6, the regression coefficients of regulating production behavior to rate of eligible agricultural products and quality level of agricultural products are, respectively (*β* = 0.463, *P* < 0.01) (*β* = 0.684, *P* < 0.01) (*β* = 0.628, *P* < 0.01) (*β* = 0.678, *P* < 0.01), government restraint factors to rate of eligible agricultural products and quality level of agricultural products are, respectively (*β* = 0.361, *P* < 0.01) (*β* = 0.099, *P* < 0.01), but the regression coefficient decreases (59). The result indicates that the farmers' green production behavior plays a partial intermediary role between government driving factors and agricultural product quality security. The discriminate coefficient F=38.6 and 30.5. In turn, the model B7, B8 are added to the model A7, A8; the model B9, B10 are added to the model A9, A10; the model B11, B12 are added to the model A11, A12; the model B13, B14 are added to the model A13, A14; the model B15, B16 are added to the model A15, A16; the model B17, B18 are added to the model A17, A18; the model B19, B20 are added to the model A19, A20, show that farmers' green production behavior plays an intermediary role between quality safety of agricultural products and driving factors. Hypothesis H7 is established. Other results of the research are shown in Table 5. The mediation effect diagram is shown in Figure 2.

## Discussion and conclusion

### Discussion

Under the normalization of COVID-19 prevention and control in China, the green production behavior of farmers is affected by many factors. The structural equation model analyzed the related data, and found that the individual characteristic driving factors, the government driving factors, the organization driving factors, the market driving factors and the social driving factors were positively related or negatively related to the farmers' green production behavior, based on the quality and safety of agricultural products. The following factors are analyzed from different driving factors.

For individual driving factors, among the individual driving factors, the age of agricultural producers is negatively related to the agricultural producers according to the production quality and safety, and the marital status is negatively related to the dependent variables and has an indirect relationship with the age. There is also a negative correlation between the cultivated area of arable land owned by agricultural producers and the quality and safety of agricultural products produced by agricultural producers. In order to save time and effort, it will rely more on the use of pesticides and high-efficiency chemical fertilizers, resulting in the quality and safety of agricultural products. The planting cost is positively correlated with the dependent variable. When the planting cost of the agricultural producer is higher, it means that the farmers will pay more attention to the quality and safety of agricultural products. In terms of individual characteristics, although the gender factor of farmers in this study did not have a certain impact on farmers' production behavior, many scholars believe that gender is an important factor affecting farmers' production behavior, and that men choose to plant safety in farmers' production behavior. The willingness to produce agricultural products is higher than that of women. Doss (60) analyzed the impact of gender on improving crop varieties and management systems in African countries, and concluded that males were more inclined to choose superior varieties than females in selecting varieties. Kishor (61) selected 325 men and 109 women in Nepal to study pesticide use knowledge and behavior, and found that men's pesticide use safety and awareness levels were higher than women's, resulting in a lower risk of pesticide application. Other experts and scholars have reached similar conclusions. Abhilash and Singh (1) found in a study of pesticide application behavior in India that farmers, without the awareness of scientific production, ignored health and environmental hazards while pursuing pest control and yield increase. Farmers' awareness affects their pesticide use behavior. Therefore, farmers' age, planting area, planting cost, gender and awareness will affect farmers' production behavior, which in turn affects the quality and safety of agricultural products.

For government driving factors, whether the government does tax on agricultural producers is negatively related to agricultural producers in accordance with the standard production quality and safety. It shows that the more serious the government's tax is, the lower the agricultural producers' willingness to standardize the production quality and safety of agricultural products. Whether the agricultural producers are punished for the uneasy production of agricultural products is positively related to the agricultural producers in accordance with the standard production quality and safety. It shows that the agricultural producers pay attention to the economic benefits. When the quality of the production of the agricultural products is not up to the standard, the agricultural producers will pay attention to the production of the quality and safety of the agricultural products. In terms of government-driven factors, although only taxes and penalties are related to farmers' production behavior in this study, some scholars have received government-designated laws, regulations and training that have an impact on farmers' production behavior. Abhilash and Singh (1) believes that in the process of farmers producing agricultural products, the government strictly implements various laws and regulations in the application of pesticides, which can play a certain regulatory role in the production, distribution and application of pesticides. The government's training of agricultural producers will play a certain driving role in the process of farmers' production of agricultural products. Hruska and Corriols (62) trained 1,200 corn farmers in Nicaragua, and in the following 2 years, the farmers' behavior and future after the training were analyzed. The behavior of trained farmers was compared and found that trained farmers used fewer pesticides, spent less on pest control, and gained greater economic benefits. Therefore, taxation, penalties, laws and regulations, and training among the government-driven factors affect farmers' production behavior, which in turn affects the quality and safety of agricultural products.

For industrial organizational driving factors, in the organization driving factors, the organization of unified training is positively related to the agricultural products of safety and safety of farmers' safety, indicating that farmers understand the relevant knowledge of the quality and safety of agricultural products, and the agricultural producers are willing to take the initiative to choose the only quality and safe agricultural products. There is a positive correlation between the rules and regulations of organizational management and the quality and safety of agricultural products produced by agricultural producers in accordance with the specifications. In terms of organizational driving factors, this study found that the rules and regulations of unified training and organizational management have a positive impact on the quality and safety of agricultural products, but some scholars have also studied that the atmosphere of civil organizations has an impact on the quality and safety of agricultural products. Rola and Pinglai (63) and other studies on the impact of pesticide use on rice yield and farmers' health believe that improving farmers' experience and lessons through training will enable farmers to more rationally assess yield losses related to pests and diseases. Therefore, farmers are encouraged to use fewer pesticides, improve rice safety and protect farmer's health and the quality and safety of agricultural products. Once the advanced cooperative management culture is formed, it will arouse the strong interest of farmers in learning, and can effectively improve the production skills and comprehensive quality of farmers, thereby mobilizing farmers' initiative, enthusiasm and creativity, and encouraging farmers to implement green production behaviors (64, 65). Therefore, organizational training and organizational atmosphere affect the quality and safety of agricultural products by affecting farmers' green fertilization behavior.

For Market driving factors, the market supervision of agricultural product quality is positively correlated with the production of quality and safe agricultural products by agricultural producers according to regulations, indicating that the greater the market supervision is, the more farmers will regulate their own production behavior, and the higher the quality and safety of agricultural products produced. The smooth sales channels of high-quality agricultural products are positively correlated with the production of quality and safe agricultural products, indicating that agricultural producers pay attention to the sales channels of safe agricultural products. When safe agricultural products have an advantage in sales channels, farmers are willing to produce quality and safe agricultural products. In terms of market driving factors, another scholar, Wollni, found that since participating in the organic market can improve farmers' returns, the market has a promoting effect on the increase of investment in conservation agriculture and can promote farmers to carry out green production. Burton R believes that profitable agricultural environmental protection programs may be more effective in changing farmers' behavior in the long run. Illukpitiya and Gopalakrishnan (32) believes that there is a positive correlation between farmers' income and farmers' land protection behavior. The higher the farmers' farming income, the higher the price of high-quality agricultural products given by the market, and the more willing they are to invest more in land protection to obtain high-quality agricultural products. Therefore, the market supervision of agricultural product quality, the smoothness of sales channels, market investment and sales income affect farmers' green production behavior and thus affect the quality and safety of agricultural products.

For Social driving factors, there is a negative correlation between public opinion and agricultural producers' production of quality and safe agricultural products according to specifications, indicating that agricultural producers do not choose to produce quality and safe agricultural products because of public opinion on safe agricultural products. The online publicity of the quality and safety of agricultural products is positively correlated with the production of quality and safe agricultural products by agricultural producers according to specifications, indicating that the media publicity has a greater impact on agricultural producers' choice of producing quality and safe agricultural products, and the media's influence on agricultural producers is far greater than that of the public. Public opinion on safe agricultural products. At the same time, the impact of agricultural extension services on green production behavior, and agricultural extension services will have an impact on the adoption of agricultural technologies. Once high-tech is adopted, it will improve agricultural productivity and reduce the intake of harmful substances to a certain extent (34). When the social non-agricultural labor market is very developed, farmers are more confident in facing agricultural risks, so farmers will increase the input and application amount of chemical fertilizers and pesticides, which will cause a greater burden on cultivated land, and the residues of pesticides and fertilizers are harmful to health (66). To sum up, the development of public opinion, media publicity, agricultural extension services and the non-agricultural labor market will affect farmers' green production behavior, which in turn affects the quality and safety of agricultural products.

### Conclusion

Integrated consideration of the normalization of COVID-19 prevention and control as a public health event. Through the ternary interaction theory, the green production behavior of farmers is studied, and the driving factor model of agricultural product quality and safety is constructed. Qualitative research shows that farmers' green production behavior has a positive impact on the quality and safety of agricultural products. Farmers' characteristic factors, government-driven factors, industrial organization-driven factors, market-driven factors and social-driven factors have a positive impact on farmers' green production behavior. The green production behavior of farmers plays an intermediary role between the quality and safety of agricultural products and the driving factors. Quantitative research shows that age, marital status and planting area among farmers' characteristic factors are negatively correlated with farmers' green production behavior. The younger, unmarried, and small farmers pay more attention to green production behavior, which will promote the improvement of the quality and safety of agricultural products; Among the government driving factors, the taxation of agricultural producers is negatively correlated with farmers' green production behavior, that is, the more government tax, the lower the willingness of farmers to green production behavior, which is not conducive to the improvement of agricultural product quality and safety. Punishment of farmers who produce inferior agricultural products is positively related to farmers' green production behavior, that is, punishment will regulate farmers' production behavior, promote their green production, and help improve the quality and safety of agricultural products; The organizational unified training and organizational management rules and regulations in the driving factors of industrial organization are positively correlated with farmers' green production behavior. That is, the training enables farmers to understand the relevant knowledge of the quality and safety of agricultural products, and the rules and regulations of organization and management regulate farmers' production behavior. Promote the quality and safety of agricultural products by improving their willingness and behavioral norms for green production; Among the market driving factors, market supervision and smooth sales channels of high-quality agricultural products are positively correlated with the quality and safety of agricultural products. That is, the more formal and smooth the sales channels of agricultural products are under market supervision, the higher the willingness of farmers to choose green production behaviors, and the improvement of the quality and safety of agricultural products; Among the social driving factors, public opinion is negatively correlated with the quality and safety of agricultural products, that is, the more negative public opinions on the safety of agricultural products, the less farmers are willing to carry out green production, and the quality of agricultural products will decrease. There is a positive correlation between online publicity and the quality and safety of agricultural products, that is, the more media publicity at the social level, the more willing farmers are to carry out green production to improve the quality and safety of agricultural products. Based on the above conclusions, it can be seen that improving the quality and safety of agricultural products requires the society, the government, the market, and organizations to jointly drive the evolution of farmers from non-green to green production behaviors. It is necessary to establish a multi-agent-driven strategy under the normalization of COVID-19 prevention and control so that farmers can evolve from non-green production behavior to green production behavior from multiple perspectives and levels. Specific recommendations are as follows:

The COVID-19 outbreak is the most serious public health emergency China has suffered. It is necessary to treat the epidemic from the perspective of protecting national security and stabilizing social development and to recognize the importance of establishing a guarantee mechanism for agricultural products in public health emergencies. Therefore, a multi-subject driving mechanism for farmers' production behavior based on the quality and safety of agricultural products should be established to improve the enthusiasm and enthusiasm of agricultural producers to produce high-quality and safe agricultural products. Encourage active learning of the relevant knowledge and advanced technologies of safe agricultural products, enhance the understanding of standardized production of agricultural products with quality and safety, and improve the safety awareness of standardized production of agricultural products. The government should strengthen the publicity and education of relevant parties and the supervision of the quality and safety of agricultural products. For the government, first of all, it should undertake the responsibility of agricultural product safety education. Increase the popularization and publicity of agricultural product safety knowledge, and provide agricultural producers with corresponding knowledge or skill training on pesticide and chemical fertilizer application on a regular basis. In order to improve agricultural producers' understanding of the risks that different pesticide and chemical fertilizer application methods may cause to the safety of agricultural products, and to enhance agricultural producers' awareness of safe production. Let agricultural producers clearly know that frequent or large application of pesticides and fertilizers will cause pesticide residues and farmland pollution, and clearly understand what quality and safe agricultural products are produced. Secondly, strengthen the supervision of the circulation links under the normalization of COVID-19 prevention and control, strictly enforce the law, and establish a corresponding incentive mechanism. So that agricultural producers can earn real benefits from the production of quality and safe agricultural products in accordance with the specifications. Thereby reducing the randomness of agricultural producers in producing agricultural products at the source, and guiding agricultural producers to scientifically produce quality and safe agricultural products. Farmers should take the initiative to strengthen the awareness of producing quality and safe agricultural products. For agricultural producers, they should actively learn the application knowledge of various pesticides and chemical fertilizers and receive relevant skills training. In order to continuously improve their own cultural quality and scientific and technological level, enhance the understanding of the standardized production of quality and safe agricultural products, and reduce the risk of agricultural production safety. In addition, it can also expand its own external environmental conditions through cooperation and alliances, and enhance its ability to respond to the market. And try to obtain comprehensive and accurate agricultural product market information from various formal channels to improve their decision-making level and management ability. So as to occupy a favorable position in the agricultural product market. Mobilize the community's attention to the quality and safety of agricultural products. In this way, the society can effectively supervise the quality and safety of agricultural products, so that agricultural products with quality and safety problems can be discovered and dealt with in a timely manner. To sum up, in order to promote the development of green agriculture, the government should take the lead in increasing integration of technology, demonstration and promotion, and personnel training. And accelerating the popularization of several advanced and applicable green agricultural technologies in the field of agricultural production, to ensure that green production methods can be truly implemented and achieve sustainable development. Guided by market demand, the increase of green and high-quality agricultural products shall be placed in a prominent position, the industrial structure reform shall be promoted, and the consumption needs of the people for safety, quality, nutrition, and health shall be met. Use green agricultural machinery to integrate the concept of agricultural green development into all aspects of agricultural production and operation. Therefore, to ensure the quality and safety of agricultural products under the normalization of COVID-19 prevention and control, it is necessary to establish a multi-agent driven mechanism for farmers' green production behavior.

### Research limitations

There are only three provinces where the representative objects selected in this study are located, which has certain limitations. Based on this research, the research sample can be further expanded to study the driving factors of farmers' green production behavior covering different types of arable land and different crop types and the driving factors of farmers' green production behavior in other provinces in China. To explore the driving factors of farmers' green production behaviors and their driving models for the quality and safety of agricultural products in a wider range of different arable land types and different regions. It provides a useful reference for improving the quality of arable land, agricultural products and promoting sustainable agricultural development in China.

## Data availability statement

The original contributions presented in the study are included in the article/supplementary material, further inquiries can be directed to the corresponding author/s.

## Ethics statement

Ethical review and approval was not required for the study on human participants in accordance with the local legislation and institutional requirements. Written informed consent for participation was not required for this study in accordance with the national legislation and the institutional requirements.

## Author contributions

MZ and XG: Conceptualization and methodology. YT and BP: Data curation and formal analysis. YT and XG: Writing—original draft. YT, BP, MZ, and XG: Writing—review & editing. All authors have read and agreed to the published version of the manuscript.

## Funding

This study was funded by the Heilongjiang Province Postdoctoral Funding Project “Research on the Mechanism of the Influence of the Management Factors of the Canonical Form Farmer Specialized Cooperatives on the Transition in Farmer Safety Production Behavior, grant number LBH-Z17018, Ministry of Education Humanities and Social Sciences Research Fund Project Production Behavior Based on the Quality and Safety of Agricultural Products: Influencing Factors, Co-governing Mechanisms and Guiding Strategies, grant number 18YJC630162, Young Talents of Northeast Agricultural University, grant number 20XG07, The Open Project of the Key Laboratory of Modern Agricultural Equipment and Technology in the Northern Cold Region, grant number KF18-01, a general project of Heilongjiang Province Philosophy and Social Science Research Planning Project Research on Multiple Dynamic Mechanisms and Optimization Strategies of Black Soil Protection in Northeast China Based on Total Quality Management and Marketization, grant number 21JYD273, and General Project of National Social Science Foundation of China. Research on the Connection Path between Small Farmers and Modern Agriculture from the Comparative Perspective of Organization and Marketization, grant number 20BJY135, Heilongjiang Social Science Fund Project Research on Land Trust Risk Management in Heilongjiang Province based on Multiple Cogovernance, grant number 21JYB150.

## Conflict of interest

The authors declare that the research was conducted in the absence of any commercial or financial relationships that could be construed as a potential conflict of interest.

## Publisher's note

All claims expressed in this article are solely those of the authors and do not necessarily represent those of their affiliated organizations, or those of the publisher, the editors and the reviewers. Any product that may be evaluated in this article, or claim that may be made by its manufacturer, is not guaranteed or endorsed by the publisher.
